# Evaluation of left atrial myocardial deformation in patients with acute MR after STEMI using CMR feature tracking

**DOI:** 10.1186/1532-429X-18-S1-P64

**Published:** 2016-01-27

**Authors:** Tomas Lapinskas, Laura Urbonaite, Paulius Bucius, Augustinas P Fedaravicius, Agnieta Stabinskaite, Marta Ejsmont, Antanas Jankauskas, Remigijus Zaliunas

**Affiliations:** 1 Department of Cardiology, Lithuanian University of Health Sciences, Kaunas, Lithuania; 2 Faculty of Medicine, Lithuanian University of Health Sciences, Kaunas, Lithuania; 3 Department of Radiology, Lithuanian University of Health Sciences, Kaunas, Lithuania

## Background

Left atrium (LA) is an important predictor of cardiovascular morbidity and mortality. Data related to LA functional changes during acute MR after ST-segment elevation myocardial infarction (STEMI) are limited. CMR is rapidly evolving imaging modality and feature tracking becomes very promising technique for assessment of myocardial deformation. The aim of this study was to investigate LA functional changes during acute MR in patients with STEMI using cardiac magnetic resonance (CMR) feature tracking.

## Methods

A total of 30 participants (mean age 59 years; 70% male) were enrolled into the study and underwent CMR at 1.5 Tesla (Siemens Magnetom Aera). LA volumetric and myocardial deformation parameters were obtained from two- and four-chamber b-SSFP cine images. LA strain (passive strain (ε_e_), corresponding to atrial conduit phase, active strain (ε_a_), corresponding to atrial booster pump phase and total strain (ε_s_), corresponding to atrial reservoir phase) and SR (peak positive strain rate (SRs), corresponding to atrial reservoir phase, peak early negative strain rate (SRe), corresponding to atrial conduit phase and peak late negative strain rate (SRa), corresponding to atrial booster pump phase) were calculated.

## Results

All LA strain parameters were significantly increased in mild MR patients, but not in moderate MR or controls (total strain (ε_s_): mild MR 34.1% ± 6.6, moderate MR 25.7% ± 6.7, controls 25.0% ± 8.1, p < 0.01; passive strain (ε_e_): mild MR 18.1% ± 3.8, moderate MR 13.8% ± 5.5, controls 12.6% ± 5.7, p < 0.05; and active strain (ε_a_): mild MR 16.0% ± 4.7, moderate MR 11.8% ± 3.7, controls 12.3% ± 3.2, p < 0.05). LA strain rate parameters did not reach statistical significance. Intraclass correlation coefficient analysis revealed strong interobserver agreement for all LA strain and strain rate parameters.

## Conclusions

LA longitudinal deformation is enhanced during acute MR in patients with STEMI, but only when MR is mild. CMR feature tracking is highly reproducible, less time consuming and potentially valuable tool for clinical and research applications.

**Table 1:** Comparison of LA strain (ε) and strain rate (SR) parameters MR population and controls

		Controls (n = 10)	Mild MR (n = 10)	Moderate MR (n = 10)	P value
Left atrial function	Left atrial strain (%)				
Reservoir	εs	25.0 (8.1)	34.1 (6.6)	25.7 (6.7)	0.009
Conduit	εe	12.6 (5.7)	18.1 (3.8)	13.8 (5.5)	0.026
Booster pump	εa	12.3 (3.2)	16.0 (4.7)	11.8 (3.7)	0.041
	Left atrial strain rate (s-1)				
Reservoir	SRs	1.1 (0.2)	1.2 (0.1)	1.0 (0.2)	0.093
Conduit	SRe	-0.5 (0.2)	-0.7 (0.1)	-0.6 (0.2)	0.104
Booster pump	SRa	-1.0 (0.2)	-1.1 (0.4)	-0.9 (0.3)	0.738

ε, strain; SR, strain rate; MR, mitral regurgitation. Bold p values indicate a significance level <0.05.

